# Blood–Brain Barrier Changes and Related Microvascular Outcomes in Long-COVID: A Comprehensive Review

**DOI:** 10.3390/life16081227

**Published:** 2026-07-24

**Authors:** Marcella Chagas-Sena, Wei Ling Lau, Thomas Edward Lane, Paola Cristina Resende, Ane Claudia Fernandes Nunes

**Affiliations:** 1 Nova Friburgo Health Institute, Fluminense Federal University, Nova Friburgo 28625-650, Brazil; 2 Division of Nephrology, Department of Medicine, University of California, Irvine, CA 92868, USA; 3 Department of Neurobiology & Behavior, University of California, Irvine, CA 92697, USA; 4 Laboratory of Respiratory, Exanthematous, Enteric Viruses and Viral Emergencies, Oswaldo Cruz Institute, Rio De Janeiro 21040-900, Brazil; 5 Laboratory of Cellular, Genetic, and Molecular Nephrology, Renal Division, Faculty of Medicine, University of São Paulo, São Paulo 01246-903, Brazil

**Keywords:** SARS-CoV-2, Long-COVID, Blood–Brain Barrier (BBB), Post-Acute Sequelae of COVID-19 (PASC)

## Abstract

Caused by the SARS-CoV-2 virus, the COVID-19 pandemic is still considered a complex challenge, with manifestations not only of respiratory issues, but also conditions related to chronic cerebrovascular damage. Endothelial biomarkers, neuropathological and neuroimaging findings indicate endothelial dysfunction, microthrombosis, and disruption of the blood–brain barrier (BBB) are central mechanisms for acute and chronic ischemic and hemorrhagic cerebral events. Understanding these mechanisms is vital to reducing their impact on population health, whether through treatment or prevention of adverse outcomes. The objective of this study is to perform a review of the scientific literature on the post-infection effects of SARS-CoV-2 affecting the cerebral endothelium and the BBB, correlating them with potential clinical outcomes. Material and Methods: Analysis of studies extracted from the PubMed database using the following terms: Long-COVID “AND” SARS-CoV-2 “AND” blood–brain barrier. Inclusion criteria: keywords, publications related to the topic, and primary studies published after peer review. Exclusion criteria: preprint studies, publication outside of the timeframe 2020–2025, study design not compatible with this research, and full text not available. Results: An initial 121 studies were identified, of which 105 were excluded due to not meeting all inclusion criteria and 6 studies were inaccessible due to not being in the English language and full-text access limitations. Fifteen articles were included in the analysis, for topics as expression of viral receptors in the endothelium, markers of their activation, cerebral microvascular injury and coagulopathies. To clarify the pathogenic cascade, the evidence was stratified by biological model where in vitro evidence demonstrates that the Spike protein induces direct endothelial toxicity and platelet aggregation, establishing the primary molecular insult. Animal models confirm the translation of this insult into structural degradation of the BBB and pericyte loss. Clinically, infection-phase findings, characterized by multifocal microthrombosis and permeability spikes, act as the determining event that predisposes to the persistent neuroinflammatory environment. Biomarkers of BBB disruption and neuronal damage were consistently reported, with persistence of BBB dysfunction modifying risk stratification and rehabilitation efforts. Reported cases of Long-COVID demonstrated normalization of BBB markers without correlation with long-term symptoms, suggesting that other mechanisms are involved in Long-COVID. Conclusion: Cerebral endotheliopathies and BBB dysfunction in patients with COVID-19 continue to impact the health of the population. The scientific literature indicates that SARS-CoV-2 induces cerebral endothelial injury, BBB disruption, and an increased risk of vascular events related to endotheliopathy, inflammation, and hypercoagulability. Understanding the impact of COVID-19 pathology on the population and developing prospective studies is essential to quantify the prevalence and mechanisms of brain injury. The identification of molecular targets and infection pathways are promising toward defining both preventive and therapeutic strategies to improve outcomes in the Long-COVID population.

## 1. Introduction

SARS-CoV-2, the acronym for Severe Acute Respiratory Syndrome Coronavirus 2, emerged in late 2019, rapidly evolving from a pneumonia of unknown cause to a global health emergency [1]. Initially defined by respiratory symptoms, the disease revealed a multisystemic nature early in the pandemic, characterized by cardiac injury, renal failure, neurodegeneration, and RNAemia (RNA presence in the bloodstream) [2,3,4,5,6]. A common denominator emerged from clinical and pathological reports: endothelial damage. SARS-CoV-2 is not merely a respiratory virus but causes systemic endotheliopathy [7,8,9].

Following the infection phase, a significant proportion of patients (10–20%) fail to recover completely, developing Long-COVID or Post-Acute Sequelae of COVID-19 (PASC) [10]. This syndrome is defined by the World Health Organization (WHO) as a condition occurring in individuals with a history of probable or confirmed SARS-CoV-2 infection, typically arising three months after the onset of the disease with symptoms that last for at least two months and cannot be explained by an alternative diagnosis. These symptoms, which include fatigue, shortness of breath, and cognitive dysfunction, impact everyday functioning and may manifest as new, persistent, or fluctuating health issues over time. In this context, neurological complaints—such as brain fog, memory deficits, and dysautonomia—are among the most debilitating in this clinical picture [8,9,10,11,12,13], which could havea gatekeeper role attributed to the brain–blood barrier (BBB), since it blocks large molecules, hydrophilic compounds, and peripheral immune cells from entering the brain.

**Figure 1:**
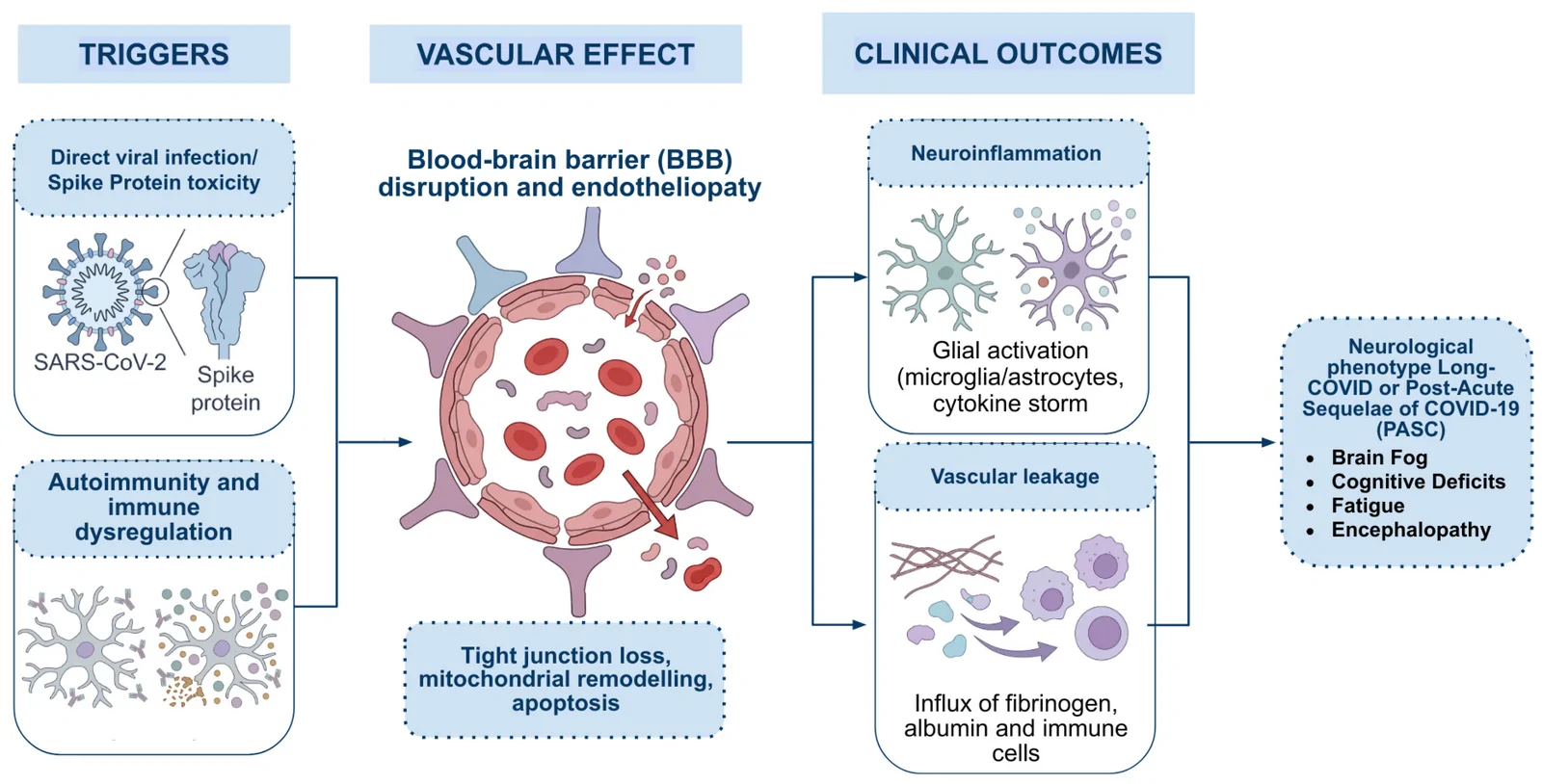
The BBB as the critical interface between systemic SARS-CoV-2 infection and neurological outcomes. This conceptual model illustrates BBB dysfunction as the pivotal mechanism bridging viral-induced systemic inflammation with the onset of chronic neurocognitive phenotypes observed in Long-COVID.

The vascular effects of COVID-19, in particular those that target the BBB, were unclear at the start of the pandemic. Several mechanisms have emerged, ranging from endothelial injury to complex vascular damage, as well as coagulation defects. At the same time, thrombo-inflammatory processes and tissue hypoxia likely also contribute to neuroinflammation in Long-COVID. These mechanisms are summarized in Figure 1.

This review explores the existing evidence of the “Vascular Hypothesis” as a unifying thesis: the vascular damage triggers a cascade of thrombo-inflammation, tissue hypoxia, and neuroinflammation that drives chronic symptoms understood nowadays as Long-COVID.

## 2. Materials and Methods

A comprehensive literature search was conducted across the PubMed database focusing on three key terms ‘Long-COVID,’ ‘SARS-CoV-2,’ and ‘blood-brain barrier.’ Inclusion criteria encompassed studies published between 1 January 2020 and 31 December 2025. The screening was performed during January 2026, which covers only studies published up to that time. Conversely, the exclusion criteria eliminated articles focused on treatment or clinical management, studies addressing only acute COVID-19 without specific reference to PASC, and research investigating outcomes related to pre-existing neurological conditions (such as Alzheimer’s disease and other dementias), psychiatric disorders, or oncological history. Studies involving pediatric populations were also omitted.

Although not a systematic review, we adopted inclusion and exclusion criteria for conducting this study in a serial analysis that was carried out independently by the authors. The first screening stage was performed by MCS and reviewed, in a blinded manner, by WLL and ACFN independently. Although we did not have any disagreement in the selection of studies, data that were potentially incompatible with the inclusion criteria were discussed among the authors to reach a consensus. The other authors, TEL and PCR, participated freely in the data analysis stage, contributing to the discussion and conclusions due to their expertise in studies on COVID-19 from the pandemic to the present day.

## 3. Results

**Figure 2:**
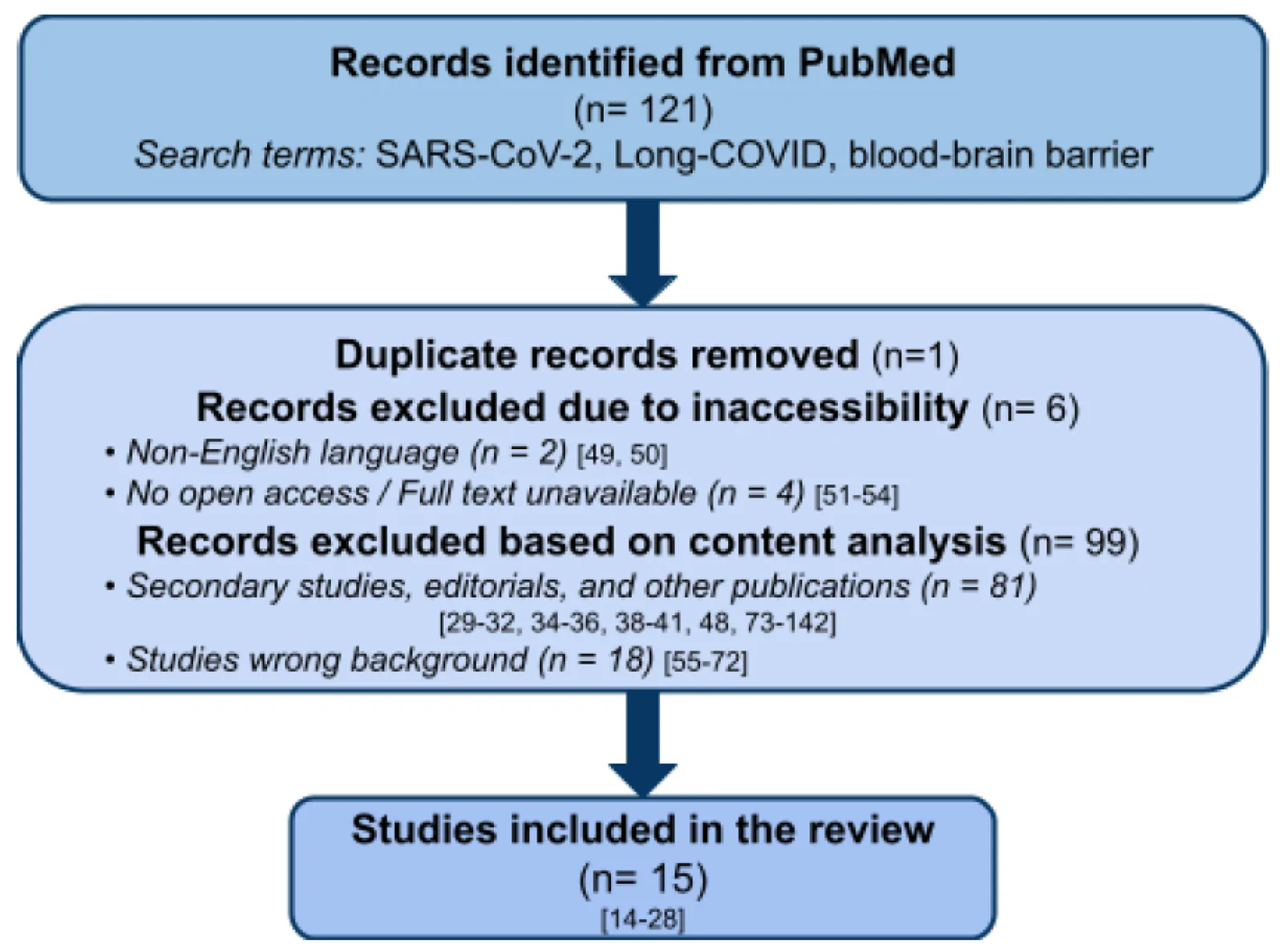
**Literature search and study selection workflow.** A total of 121 records were identified through the PubMed database search using the terms “SARS-CoV-2”, “Long COVID”, and “blood–brain barrier”. After removing one duplicate, 120 records were screened. Six records were excluded due to inaccessibility (non-English language or lack of open access), and 99 were excluded based on title and abstract review (secondary studies, editorials, or unrelated background). Finally, 15 studies met the inclusion criteria and were included in this review.

The initial search identified 121 records; after removing one duplicate record, 120 articles remained for the next screening stage, all of which were included in the list of bibliographic references. Six records were excluded due to accessibility issues, comprising two non-English articles and four for which the full text was unavailable. An additional 99 articles did not fulfil all selection criteria, thus a total of 15 articles were included in the final analysis, especially in the Discussion where other articles were used to support the argument. Screening and selection methods applied in this study are summarized in Figure 2.

Based on the inclusion criteria of the studies selected for this review, the study converges on the published literature examining BBB dysfunction and other secondary outcomes related to vascular dysregulation as central mechanisms in the pathogenesis of COVID-19 neurological complications. Due to the heterogeneity of the studies found, different approaches could be adopted throughout this comprehensive review. This was also the reason why it was not possible to design a systematic review within the standard guidelines. To make consulting the text easier, these data were even grouped into different tables. For example, in a mouse model of COVID-19 infection, Qiao et al. (2024) described BBB microvascular damage including astrocyte activation, pericyte loss, and impaired tight junctions. On the other hand, clinical investigations conducted by Bonetto et al. (2022) and Chaganti et al. (2025) confirmed that BBB disruption markers are significantly elevated in COVID-19 infection and correlate directly with the severity of the infection, suggesting that vascular stabilization could be a crucial therapeutic target to mitigate long-term damage [14,15,16].

At the molecular and cellular levels, key injury mechanisms are indirect and independent of active viral replication within the central nervous system (CNS). Motta et al. (2023) and Coelho et al. (2025) demonstrated in experimental models that mere exposure to the Spike protein or viral presence alters BBB permeability, dysregulates the bradykinin receptor via the ACE2 pathway, and induces mitochondrial remodelling and endothelial apoptosis [17,18]. Consistent with these findings, Erickson et al. (2023) observed in Expi293F suspension cells that even non-replicating viral particles can penetrate the BBB and incite neuroinflammation, with SARS-CoV-2 variants, such as Delta and Omicron, showing greater efficacy in this traversal [19]. This pathophysiology is corroborated by Zingaropoli et al. (2022), who noted in patients that neurological damage (measured by neurofilament light chain, NfL) with the severity of systemic infection, rather than direct viral invasion detectable in the cerebrospinal fluid (CSF) [20].

Analysis of CSF and immunological profiles in retrospective cohorts reveal a scenario of persistent inflammation. The identification of BBB dysfunction and elevated levels of total proteins were frequent findings, often accompanied by sustained levels of inflammatory cytokines (Jarius et al., 2022; Boesl et al., 2024) [21,22]. In their cross-sectional clinical study, Etter and colleagues (2022) described a pathological triad composed of impaired BBB, microglia activation, and a polyclonal B-cell response [23].

In this immunological context, Almulla et al. (2024) highlight the role of autoimmunity in the development of affective symptoms and chronic fatigue, while Spatola et al. (2023) suggest that an antibody response skewed toward common coronaviruses may impede complete viral clearance, perpetuating neuroinflammation [24,25].

Regarding vascular integrity, Wallensten et al. (2024) observed that while BBB permeability increases during infection, this phenomenon may be transient, returning to baseline levels after one year [26]. On the other hand, the transient nature of BBB disruption contrasts with the severity of acute encephalopathy, described by Atluri et al. (2021), as an example of direct neurological impacts; it demonstrates that CNS dysfunction can occur alongside or independently of sustained barrier leakage, which raises questions regarding the chronicity of symptoms reported, similar to described by Guasp et al. (2022), who emphasize neuronal damage as the primary functional outcome in patients with Long-COVID [27,28].

Etter et al. (2022) [23], Spatola et al. (2023) [25], and Wallensten et al. (2024) [26] reported that the patients included in their studies had not been vaccinated against SARS-CoV-2 because recruitment was carried out before COVID-19 vaccines became available.

The main findings from the articles that used human samples grouped in this review are compiled in Table 1.

In experimental animal or cellular models, vaccination status does not apply or was not included as a variable in the design of laboratory studies. In other studies, vaccination information was not reported or was not included in the selection and description criteria for participants in the original articles.

The main characteristics of studies using animal models are summarized in Table 2, and cellular models are highlighted in Table 3. Biological source and biomarkers of BBB dysfunction and neuroinflammation are presented in Table 4.

In Long-COVID, the literature indicates that this thromboinflammatory phenotype and vascular dysregulation often do not resolve completely. This sustained neurovascular compromise and resulting local hypoxia form the biological basis for long-term symptoms such as chronic fatigue and brain fog, persisting even after stabilization of classic markers of acute barrier leakage.

## 4. Discussion

In this comprehensive review, our primary finding indicates that BBB dysfunction and chronic endotheliopathy can represent an important role bridging the SARS-CoV-2 infection with the persistent neurocognitive sequelae of Long-COVID. As secondary findings, the synthesized literature demonstrates that this barrier impairment is largely independent of direct viral replication within the CNS. Instead, the results suggest that the systemic immune dysregulation, autoimmunity, and Spike protein toxicity could be relevant triggers. Consequently, this sustained endothelial failure seems to initiate a sequential cascade of chronic neuroinflammation, glymphatic clearance failure, and ongoing neuro-axonal damage.

**Figure 3:**
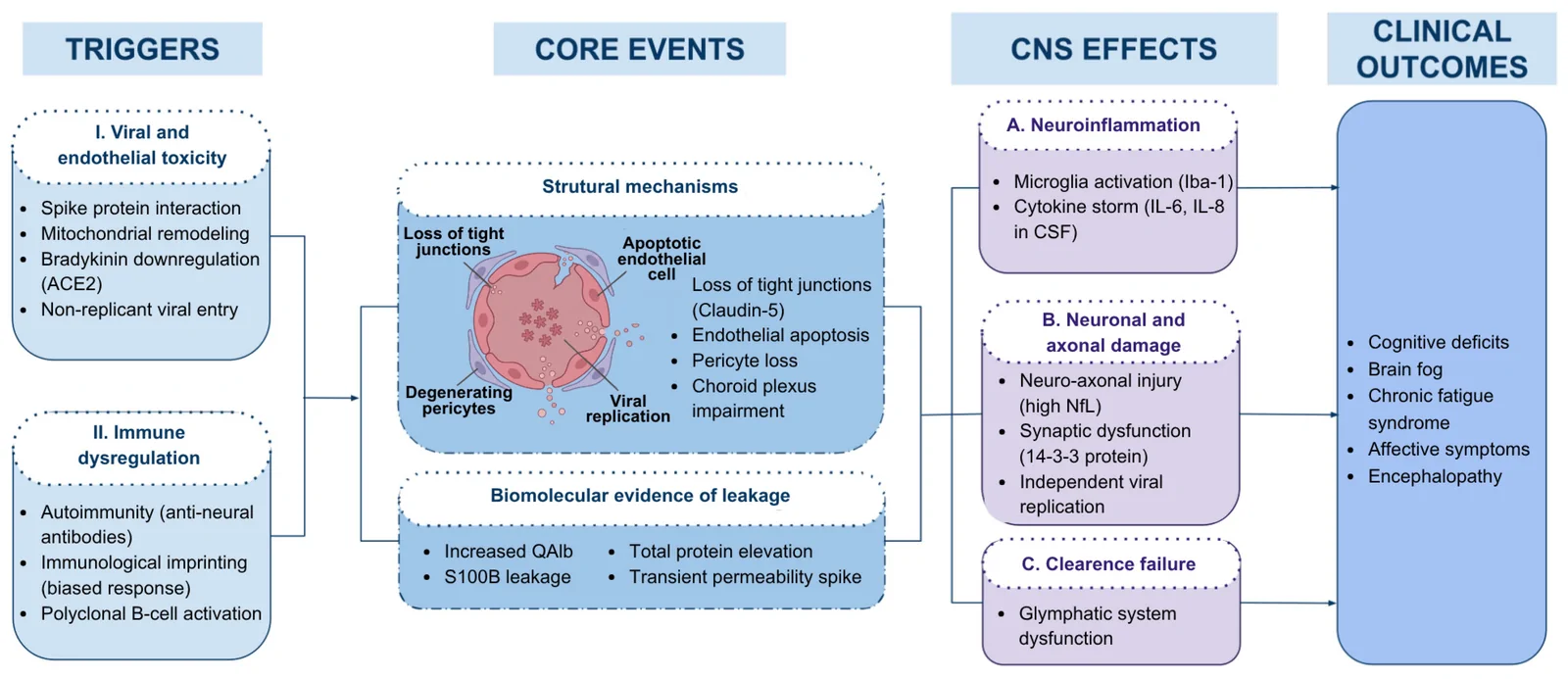
Integrated pathophysiology of Neuro-PASC. This diagram synthesizes the converging pathways of cerebral vascular injury by SARS-CoV-2 from endothelial trigger to clinical phenotype. The cascade initiates with viral interaction and endothelial activation, including viral replication and oxidative mitochondrial remodelling. It illustrates the subsequent downregulation of tight junction proteins and pericyte dropout, events that compromise BBB integrity. This pathway facilitates the influx of neurotoxic plasma proteins (e.g., fibrinogen), culminating in sustained neuroinflammation and neuronal injury, understood here as the biological basis for persistent cognitive symptoms. The evaluation of the biomarker profile investigates three core points of the pathogenesis: the initial breakdown of the barrier, driven by the loss of tight junctions, endothelial apoptosis, and pericyte dropout is validated by direct biomolecular evidence of leakage, specifically the elevation of CSF/plasma albumin quotients (QAlb), S100B leakage, and total protein elevation. The secondary effect of this vascular leakage is the establishment of a neuroinflammatory environment, which is investigated and confirmed through markers of microglial activation (such as Iba-1) and the presence of a cytokine storm within the CNS (e.g., elevated IL-6 and IL-8 in the CSF). The ultimate downstream consequence of this sustained microvascular insult is tissue damage, objectively quantified by biomarkers of neuro-axonal injury (NfL) and synaptic dysfunction (14-3-3 protein).

To systematically evaluate the role of vascular dysregulation in Long-COVID, we assessed the biomarker profiles against a conceptual null hypothesis (H_0_) that BBB dysfunction is merely an unspecific, transient epiphenomenon of the acute SARS-CoV-2 infection, with no direct mechanistic link to the persistent neuroinflammatory and cognitive outcomes of Long-COVID. Based on the evidence found in the studies selected for this review, it seems reasonable to reject hypothesis H_0_, since impairment of the BBB may play an important role in disease progression. Figure 3 represents some fundamental aspects of BBB pathogenesis associated with Long-COVID and the main dedicated biomarkers described to date that support this hypothesis.

By specifically linking these biomarkers to the converging pathways of cerebral vascular injury, Figure 3 visually reports what the literature included in that review presents as how the initial endothelial triggers lead to the clinical phenotypes of Long-COVID, illustrating how the biomarker profiles can actively reflect the transition from vascular leakage to chronic cognitive deficits, brain fog, and fatigue.

### 4.1. Direct Endothelial Activation: Viral Infection vs. Spike Toxicity

Evidence gathered from experimental models indicates that the BBB is not merely a target of systemic inflammation, but also a compound of direct viral interaction with endothelial cells. This aspect can be observed in other studies, beyond the batch we selected for this review. For example, Sharma et al. (2021) emphasized that the hematogenous route serves as a critical pathway for neuroinvasion, where systemic viral dissemination and cytokine storms compromise BBB integrity, facilitating viral entry [29]. Yang et al. (2022) demonstrated that SARS-CoV-2 can actively infect HBMECs, which corroborates on this hypothesis in their cellular model [30].

Conversely, it is important to note that active viral replication within the CNS is not a prerequisite for BBB injury. The viral–endothelial interaction instigates a maladaptive metabolic phenotype even in the absence of productive replication. Motta et al. (2023) demonstrated that SARS-CoV-2 exposure in HBMECs triggers an inflammatory cascade through the non-canonical NF-κB pathway, inducing mitochondrial remodeling [17]. This metabolic alteration mirrors the Senescence-Associated Secretory Phenotype (SASP, version 31), leading to endothelial apoptosis, even in the absence of productive viral replication, suggesting that the mere presence of viral components is sufficient to compromise endothelial viability. This hypothesis of “toxicity without replication” is further corroborated by Erickson et al. (2023), who observed in murine models that the S1 subunit of the Spike protein and non-replicating viral particles can cross the BBB, initiating neuroinflammation [19]. Their findings also highlighted a permeability dependent on the SARS-CoV-2 variant, where Delta and Omicron exhibited different efficiencies in crossing the barrier and inducing glial activation.

Regardless of whether the trigger is the whole virus or the Spike protein, the potential outcome looks like a disruptive metabolic phenotype that sets the stage for the molecular dismantling of the BBB.

### 4.2. Molecular Dynamics of Disruption: The ACE2–Bradykinin Axis

BBB disruption is not a single event, but a progressive cascade driven by specific enzymatic dysfunctions. The primary event in this pathophysiology is ACE2 suppression. Under normal conditions, as described in the general literature, this enzyme acts as a metabolic “brake”, degrading active kinins [30]. However, Coelho et al. (2025) elucidated that SARS-CoV-2 infection induces downregulation of ACE2 on the cell surface, potentially weakening this layer [18].

### 4.3. The Biochemical Trigger: Bradykinin Storm

Biochemically validating the bradykinin storm theory, the direct consequence of this enzymatic loss is the inability to degrade Des-Arg9-Bradykinin (DABK) [18]. Unlike constitutive receptors, pathological signaling occurs through the Bradykinin B1 Receptor (B1R), which is inducible and has a high specific affinity for accumulated DABK, in agreement with general conceptual data [31]. In the inflammatory scenario of COVID-19, as demonstrated by Coelho et al., ligand-receptor binding triggers a potent intracellular cascade characterized by Gq activation, a sudden increase in cytosolic calcium ([Ca2+]i), and sustained nitric oxide (NO) production. This pathway promotes extreme vasodilation and creates a local cytotoxic environment [14,18].

### 4.4. Structural Collapse: Claudin-5 and Pericytes

The biochemical storm appears to ultimately target the physical structure of the barrier. In this context, the main target is Claudin-5, the tetraspanin protein fundamental to the tight junctions of the BBB, which is a generally accepted conceptual idea [32]. In this sense, in accordance with a stressed and infectious cellular environment of COVID-19, these molecules can form a kind of “zipper” that restricts paracellular passage. The relevant vascular outcome then occurs when B1 receptor signaling intersects with structural integrity. In vivo evidence confirms that sustained calcium signaling and actin cytoskeleton reorganization force endocytosis or degradation of Claudin-5 [18], which can be corroborated by the biochemical analysis described in 2021 by Karamyan, V.T. [31], in a study beyond this review. Simultaneously, Qiao and colleagues provided crucial in vivo confirmation of the loss of pericytes—the cells that provide mechanical support to capillaries [14]. Thus, without Claudin-5 anchored to the membrane and without the support of pericytes, the barrier suffers a significant loss in its integrity. The result is the uncontrolled leakage of plasma components into the parenchyma, establishing edema, which may be directly associated with the severe tissue damage observed in Neuro-COVID [14,30].

### 4.5. Systemic Immune Aggravation and Thromboinflammation

From a pathological point of view, thromboinflammation can be summarized as a bidirectional process between blood coagulation, due to thrombosis, and the immune system, due to inflammation. In this context, inflammatory responses trigger excessive coagulation, while activated coagulation factors further amplify inflammation, leading to severe vascular damage, tissue injury, and potential organ failure. In the course of COVID-19 events, the transition from described endothelial injury to generalized neurological dysfunction is mediated by a systemic hyperinflammatory state that evolves into a thromboinflammatory phenotype. Corroborating this hypothesis, some studies selected in this review elucidate how peripheral immune dysregulation can be translated into CNS pathology [14,15].

### 4.6. Immunologic Imprinting

The persistence of inflammation in Long-COVID is not random, but appears to be driven by specific immunological profiles. Spatola et al. (2023) introduced the crucial concept of “immunological imprinting,” demonstrating that neurological outcomes are shaped by prior exposure to other coronaviruses [25]. This biased immune response is not sufficient to efficiently eliminate the virus, leading to a state of chronic activation.

Expanding on this dysregulation, some research has identified distinct characteristics in severe neuro-COVID, such as elevated levels of IL-6 and TNF-α, cytokines that could be correlated with the severity of encephalopathy [23]. Also, within the neuropsychiatric context, we can include the study by Almulla and colleagues who analyzed the relationship between chronic inflammation and specific outcomes [24]. These findings together highlight a significant elevation of autoimmune markers, specifically specific autoantibodies, in patients with Long-COVID, demonstrating that IgM-MBP (myelin basic protein) and IgG-MBP act as general predictors of the syndrome, while the presence of IgG-MOG (myelin oligodendrocyte glycoprotein) correlates with Myalgic Encephalomyelitis/Chronic Fatigue Syndrome (ME/CFS). Following this line of thought, antibodies such as IgM-synapsin and IgA-MBP have been identified as robust predictors of affective symptoms. It appears that, along with the viral infection, this systemic autoimmune exacerbation acts as a primary trigger for additional neurological consequences, which we illustrate in Figure 3 [24]. This distinct peripheral pathway may, in part, explain the unique nature of Long-COVID syndrome; for example, while these autoantibodies circulate chronically in the blood, studies on the acute phase of infection have found a complete absence of antineuronal antibodies locally in the CSF [28]. Considering these aspects, the continuous neurological manifestations in Long-COVID are better characterized not by a localized intrathecal response, but by a continuous systemic autoimmune dysfunction that targets CNS proteins from the periphery [24,25].

### 4.7. Systemic and Cns Inflammation

A key finding that emerged among the studies selected in this review as a prognostic hypothesis was that widespread systemic inflammation may be a determining factor in neuroinflammation. Bonetto and colleagues (2021) and Zingaropoli et al. (2022) independently confirmed that plasma biomarkers of BBB disruption (S100B, MMP-9) and neuronal damage (NfL) are significantly elevated and correlated with the severity of systemic disease. These findings support the concept that a “peripheral storm” can directly attack the neurovascular unit [15,20].

This hypothesis can also be considered based on the studies by Jarius et al. (2022) and Boesl et al. (2024), which report that patients with COVID-19 do not manifest classic viral encephalitis [21,22]. On the other hand, both studies found that although many patients present with BBB disruption (elevated CSF albumin quotient, QAlb), there is an absence of CSF pleocytosis. Boesl et al. (2024), in particular, argue that this points to a “cytokine encephalopathy” rather than direct viral replication in the CNS [22NfL]. The damage may be mediated by soluble factors (cytokines, fibrinogen, or others) that cross the damaged barrier, rather than T cell infiltration [21,24].

This barrier leakage may have a temporal dimension, which can be supported by the study by Wallensten et al. (2024), where they observe that although NfL and glial activation markers (GFAp) peak at 4 months, they tend to normalize at 12 months in many patients [26]. However, the persistence of symptoms in the absence of continuous elevation of the biomarker suggests that the initial “impact” may trigger subsequent neurodegenerative processes that become self-sustaining.

### 4.8. Thromboinflammation and Hypoxia as Functional Outcomes

The biochemical environment described by the Etter and Bonetto groups (elevated cytokines, MMP-9) creates the perfect substrate for thromboinflammation [15,23]. Another aspect to be considered was studied by Atluri et al. (2021) who focused on the clinical presentation of encephalopathy; their description of “confusional states” and “altered mental status” aligns with a possible hypoxic/metabolic etiology rather than a purely infectious one [27].

Integrating these findings that demonstrate pericyte loss and capillary destabilization with systemic data, a possibility also emerges to be considered, which is the loss of anticoagulant properties in the inflamed endothelium [14]. As a contextualizing model, acute infection data are used to elucidate the cellular mechanism of this failure in Long-COVID. It is only from this acute scenario that anatomical confirmation is obtained, through autopsies, that microthrombi and platelet aggregates physically adhere to the cerebrovascular wall, a process driven by direct endothelial activation via Spike protein [18], which can also be supported by other general studies more focused on other COVID outcomes in a broad way [33,34,35,36,37].

When transposing this established model to Long-COVID, the general literature shows that the thromboinflammatory phenotype does not resolve [38]. As visual confirmation of this platelet damage in the brain in vivo in the chronic phase is methodologically unfeasible to date, extrapolation of the acute model becomes necessary to explain the continuous systemic platelet hyperactivation observed in these patients [19]. These chronically activated platelets maintain the release of mediators that prolong the permeability of the BBB [15,20]. Adding to this mechanism, Ryu et al. (2024), proposes the molecular basis of fibrin-induced neurotoxicity, contrasting clinical data and biomarkers that support the hypothesis of an oxygen-deprived brain due to microcirculatory failure as a functional outcome [39]. In general, this “functional deviation” and persistent neurovascular impairment can sustain local hypoxia, forming the biological basis for long-term clinical findings such as “brain fog” and strong overlaps with ME/CFS, a condition historically linked to cerebral hypoperfusion and metabolic failure, as observed in the selection of this review [14], as well as in other studies [40,41].

### 4.9. Chronic Consequences: From Impaired Metabolic Clearance to Synaptic Excitotoxicity

The ultimate consequence of the neurovascular unit disruption and thromboinflammation described may establish a neurodegenerative environment. The literature selected in this review suggests that Long-COVID represents a state of accelerated biological aging and proteinopathy, driven by the failure of clearance mechanisms and synaptic excitotoxicity [16,20,24]. This aspect also emerges in another study focused on cognitive status, which is not the focus of our study, but equally important [42]. From this hypothesis, we can suggest how persistent vascular impairment translates into microcirculatory failure and stagnation of neurotoxic metabolites in the brain parenchyma.

### 4.10. From Cytokine Storm to Glymphatic Clearance Failure

A critical, yet often overlooked, mechanism linking vascular injury to neurodegeneration is the impairment of the glymphatic system. Chaganti and colleagues (2025) describe that perivascular spaces, essential for the elimination of metabolic waste, are structurally and functionally compromised in Long-COVID [16].

As glymphatic flow is driven by arterial pulsation and depends on the polarity of Aquaporin-4 (AQP4) channels in astrocyte footplates, endothelial damage and pericyte loss can compromise the “pump” of this cleaning system [14,17]. This is an important aspect, considering that AQP4 is the main water channel protein in the CNS, facilitating the bidirectional transport of water across BBBCSF interfaces to maintain osmotic balance, regulate the elimination of brain waste, and manage ionic homeostasis. Congestion of perivascular spaces can lead to stagnation of neurotoxic metabolites and inflammatory cytokines within the parenchyma.

This mechanism agrees with the hypothesis of Theoharides and Kempuraj (2023), where the activation of mast cells in these perivascular locations is presented as a physical and chemical blocking factor that can prevent the drainage of β-amyloid and tau proteins, thus promoting their pathological aggregation, in a study dedicated to the neuronal aspect of COVID [43].

### 4.11. The “Fusogen Storm”, Neurodegenerative Phenotype, and Synaptic Excitotoxicity: The Basis of Brain Fog

Although neuronal status is not the central focus of this review, this clinical condition emerges concomitantly in several studies. Two of these studies were not included in our selection, but they present interesting points for the clinical scenario. In particular, they point to the failure of elimination mechanisms as a component that can generate a physiological environment for proteinopathies. Sfera et al. (2023) suggest a “Fusogenic Storm” model, arguing that the SARS-CoV-2 Spike protein induces cell–cell fusion and intracellular calcium accumulation, thus mimicking the molecular pathology of early-stage Alzheimer’s disease (AD) [44]. This is clinically corroborated by Kirmizibekmez et al. (2025), who, using functional connectivity magnetic resonance imaging, identified disrupted neural networks in post-COVID patients that overlap with patterns observed in dementia, suggesting a phenotype of accelerated neurodegeneration [45].

In this same context, Almulla et al. (2024) demonstrate that this is not a passive degeneration, but an active autoimmune process; their detection of immunoglobulins directed against distinct CNS targets, specifically MBP, MOG, and synapsin, suggests that uneliminated cellular debris triggers a secondary autoimmune attack [24]. This “fight or flight” mechanism explains why neurodegeneration progresses even after virus elimination. The presence of elevated NfL in patient plasma serves as the definitive fluid biomarker of this ongoing axonal destruction [15,20]. Taken together, these data may contribute to understanding other potential effects of BBB fragility in Long-COVID.

Although cell death explains long-term deficits, fluctuating cognitive symptoms are better explained by synaptic dysfunction. In Long-COVID, a possible explanation would be that upregulation indicates a state of generalized glutamatergic excitotoxicity. In this context, chronic neuroinflammation and the hypoxic environment lower the threshold for neuronal activation. In response, neurons appear to upregulate AMPA receptors, leading to metabolic exhaustion and synaptic noise [23,27]. This hypothesis is also supported by the work of Fujimoto et al. (2025), a study not included in our selection because it has a different focus, but which provides an advance by visualizing a systemic upregulation of AMPA receptors (α-amino-3-hydroxy-5-methyl-4-isoxazolepropionic acid) in the brains of patients with Long-COVID [42]. This excitotoxic state, combined with the loss of synaptic plasticity markers, offers a coherent mechanistic explanation for the deficits in attention and memory retention, which can also be observed in other studies dedicated to the cognitive context of Long-COVID, which, although outside the scope of this review, deserves mention [42,46,47].

Furthermore, Reas and colleagues (2025) highlight that this vulnerability is genetically stratified; carriers of the APOE ε4 allele are less able to repair this synaptic damage, predisposing them to permanent cognitive decline [48]. In particular, this polymorphism is a known genetic risk factor for late-onset sporadic Alzheimer’s disease. Carriers have an increased risk of developing Alzheimer’s disease. This risk can be described as 10–15% for non-carriers (carriers of other APOE alleles) in a baseline risk population, 20–25% for ε4 heterozygotes, or 30–55% for ε4 homozygotes.

## 5. Study Limitations

Although the mechanisms presented are consistent, this review has limitations inherent to the currently available literature. Heterogeneity is observed in the clinical follow-up for the definition of long COVID and a frequent absence of statistical adjustments for baseline confounding factors. Furthermore, the nonspecificity of some biomarkers, which also fluctuate in response to other systemic conditions, requires caution when isolating the exclusive impact of SARS-CoV-2. It should also be noted that the clearest evidence on the structural collapse of the blood-brain barrier derives predominantly from studies with exposure to the original viral strains, which requires validation of this thromboinflammatory phenotype against subsequent variants. Finally, from a methodological point of view, the design prioritized a narrative approach, dispensing with the application of formal tools for assessing the risk of bias to the selected articles. In addition to these aspects presented, a limiting factor to be considered is also the large number of articles published on COVID-19 that could not be included due to different inclusion criteria that were not contemplated for this particular study [49,50,51,52,53,54,55,56,57,58,59,60,61,62,63,64,65,66,67,68,69,70,71,72,73,74,75,76,77,78,79,80,81,82,83,84,85,86,87,88,89,90,91,92,93,94,95,96,97,98,99,100,101,102,103,104,105,106,107,108,109,110,111,112,113,114,115,116,117,118,119,120,121,122,123,124,125,126,127,128,129,130,131,132,133,134,135,136,137,138,139,140,141,142,143]. This volume of study reflects how heterogeneous the field of post-COVID-19 pandemic research has become, especially focusing on Long-COVID. In this sense, we hope to contribute to new studies targeting specific aspects that can contribute to a better understanding of the post-pandemic effects and better management of health parameters related to COVID-19 and its various variants.

## 6. Conclusions and Perspectives

The evidence gathered in this review indicates BBB dysfunction as an important and multifaceted pathogenic mechanism in Long-COVID. We describe a continuous pathogenic cascade in which initial endothelial injury, driven by molecular imbalances, progresses to structural barrier collapse, thromboinflammation, and impaired glymphatic clearance. This sequence of events suggests that the clinical manifestations of cognitive decline and fatigue are not isolated neuronal deficits, but may be consequences of systemic vascular failure that alters the metabolic and homeostatic environment of the central nervous system.

Although the molecular pathways linking vascular injury to neurodegeneration are becoming increasingly clear, translating this knowledge into clinical practice remains a challenge. Furthermore, Long-COVID has demonstrated normalization of BBB markers without correlation with long-term symptoms, as observed in some reports, suggesting that other mechanisms are involved, underpinned by the consequences of the initial disruption rather than continuous leakage.

In this context, future research should focus on validating these biomarkers in larger cohorts and conducting rigorous clinical trials to determine whether therapeutic strategies aimed at vascular stabilization can effectively interrupt this pathological cycle and ultimately improve cognitive outcomes in Long-COVID. In particular, AQP-4, *APOE ε4*, and vascular fragility markers would be good initial candidates. On the other hand, immunity-related markers could also contribute, although they are not a priority in terms of population impact. Key emerging mechanisms in the complex interplay between BBB injury and CNS dysfunction are presented schematically in Figure 3.

## Figures and Tables

**Table 1:** Main data on blood–brain barrier dysfunction and neurological outcomes in COVID-19 in human sampling.

Author	Ref.	Year Study Design	Keywords	Findings
Almulla et al.	[24]	2025 Case–Control	Long-COVID, Neuroimmune, Chronic fatigue syndrome, Depression, Affective disorders, Oxidative stress	- Autoimmune responses play a role in the pathophysiology of Long-COVID. - There is an association between brain-directed autoimmunity and the development of affective symptoms. - A link is suggested between autoimmune responses and myalgic encephalomyelitis/chronic fatigue syndrome (ME/CFS). - Understanding these autoimmune mechanisms is crucial for managing Long-COVID and related conditions. - The authors hypothesize that the interaction between autoimmunity and neurological symptoms warrants further investigation to develop effective treatments.
Atluri, et al.	[27]	2021 Case Series	COVID-19, encephalopathy, SARS-CoV-2 virus, cognitive impairment in COVID-19	- The study could not identify infectious or metabolic sources for the patients’ encephalopathy, which raises questions about the neurological impact of COVID-19. - Potential mechanisms for neurological manifestations include the BBB disruption and systemic inflammatory responses.
Boesl et al.	[22]	2024 Retrospective Cohort	Post-COVID, Long-COVID, Cerebrospinal fluid (CSF), Blood–barrier dysfunction, Neurology, Post-acute sequelae of SARS-CoV-2 infection	- The study found that the most frequent pathological finding in the CSF from patients with neurological manifestations of Post-COVID syndrome (PCS) was elevated total protein, observed in 24% of samples. - Blood–CSF barrier dysfunction, indicated by elevated QAlb, was the second most prevalent finding, occurring in 13% of samples. - CSF analysis did not reveal any pathological findings. However, it remains valuable for ruling out other etiologies of encephalopathy.
Bonetto et al.	[15]	2022 Prospective Cohort	COVID-19, neurological damage, blood–brain barrier, inflammation, blood biomarkers, critical care	- Markers of BBB disruption were significantly elevated in COVID-19 patients, particularly those with neurological complications, indicating a potential link to disease severity. - There is a difference in levels of systemic inflammation markers between COVID-19 patients with neurological complications and those with high disease severity, suggesting distinct inflammatory responses. - Overall, the authors suggest that targeting BBB disruption may be a therapeutic approach to mitigate neurological damage during the acute phase of COVID-19 and potentially reduce long-term dysfunction.
Chaganti et al.	[16]	2025 Prospective Cohort	PASC-CI, MRI, DTI-ALPS, BBB, KTRANS	- Markers of BBB disruption are significantly elevated in COVID-19 patients, especially those with neurological complications, suggesting a link to disease severity. - Distinct inflammatory responses. - Targeting BBB disruption is suggested as a potential therapeutic approach to mitigate neurological damage.
Etter et al.	[23]	2022 Prospective Cohort	Autoimmunity, COVID-19, Post-Acute COVID19 Syndrome, SARS-CoV-2	- Severe neuro-COVID is associated with a triad of findings: impaired BBB, elevated markers of microglia activation, and a polyclonal B-cell response against self and non-self antigens. - COVID-19 patients exhibit reduced regional brain volumes linked to specific CSF parameters, with a notable finding of a non-inflammatory CSF profile despite a plasma cytokine storm. - The research establishes a connection between post-acute COVID-19 syndrome and a distinct set of CSF and plasma mediators, suggesting potential targets for intervention.
Guasp et al.	[28]	2022 Prospective Cohort	COVID-19, encephalitis, neurofilaments, neuronal antibodies, SARS-CoV-2, neuro-COVID, encephalopathy, inflammatory cytokines	- Long-term functional outcomes are more closely related to neuronal damage than to the acute neuroinflammatory process, suggesting a potential disruption of the BBB due to SARS-CoV-2 infection.
Jarius et al.	[21]	2022 Retrospective Cross-Sectional	Coronavirus disease 2019 (COVID-19), Severe acute respiratory syndrome coronavirus type 2 (SARS-CoV-2), Neurological symptoms, Lumbar puncture, Cerebrospinal fluid (CSF), Oligoclonal bands, Blood–CSF barrier, Polymerase Chain reaction (PCR), SARS-CoV-2 antibodies, Antibody index, Autoantibodies, Cytokines, Central nervous system, Encephalopathy, Encephalitis, Guillain–Barré syndrome	- Blood–CSF barrier (BCB) dysfunction was the most frequent pathological finding in the CSF of COVID-19 patients without pre-existing CNS diseases, observed in 50% of samples (median QAlb of 11.4). - Elevated total CSF protein was also common (45.8% of samples, median 65.35 mg/dL) and strongly correlated with QAlb levels. - Elevated cytokine levels in the CSF were present in significant proportion of patients, often associated with BCB dysfunction, and persisted for weeks or months. - Persistent BCB dysfunction and elevated cytokine levels are suggested to play a role in Long-COVID, indicating the need for further studies.
Spatola et al.	[25]	2023 Prospective Cohort	SARS-CoV-2; fc receptor; antibody-mediated complement activation; PASC; Long-COVID	- Compromised anti-SARS-CoV-2 responses in CSF may lead to incomplete viral clearance from the brain, contributing to persistent neuroinflammation and post-acute neurological complications of SARS-CoV-2 infection. - A bias toward specific immunity to common coronaviruses in individuals with NeuroPASC may inhibit the development of novel B-cell responses to SARS-CoV-2. - NeuroPASC individuals exhibited expanded antibody responses to other common coronaviruses, suggesting an “original antigenic sin” effect. - The lack of CSF antibody profiles from PASC individuals without neurological manifestations limits direct comparisons, suggesting a potential uniqueness of NeuroPASC responses.
Wallensten et al.	[26]	2024 Prospective cohort	blood–brain barrier, astrocytes, biomarkers, COVID-19, extracellular vesicles, longitudinal study	- COVID-19 was associated with a transient increase in BBB permeability as evidenced by elevated plasma levels of astrocyte biomarkers. - Peak biomarker levels were observed 4 months after hospitalization, but these levels returned to baseline within 12 months. - Elevated biomarker levels were not correlated with any self-reported long-term symptoms experienced by patients. - The authors suggest that further research is needed to understand the underlying mechanisms causing long-term symptoms in COVID-19 patients.
Zingaropoli et al.	[20]	2022 Prospective Cohort	neurofilament light chain; NfL; matrix metalloproteinases; MMPs; ddPCR; Long-COVID; neuro-COVID; zymography	- CNS damage and altered BBB integrity in COVID-19 patients with severe neurological symptoms were primarily correlated with COVID-19 severity rather than direct neuroinvasion by SARS-CoV-2. - CNS damage and altered BBB integrity may occur even in the absence of detectable SARS-CoV-2 RNA in CSF or in the blood. - The study supports the potential of NfL (neurofilament light chain) as a diagnostic and prognostic biomarker for neurological complications in COVID-19. - A significant number of COVID-19 patients present with neurological symptoms during the acute phase and may have neurological sequelae after recovery. - The presence of SARS-CoV-2 RNA in CSF and plasma was not associated with increased levels of NfL or MMPs in the CSF, indicating that CNS damage may occur independently of viral detection.

**Table 2:** Main data on blood–brain barrier dysfunction and neurological outcomes in COVID-19 in animal sampling.

Author	Ref.	Year	Study Design	Keywords	Findings
Erickson et al.	[19]	2023	In vivo (Mouse)	COVID-19, SARS-CoV-2, blood–brain barrier, Neuroinflammation, Alzheimer’s disease, Obesity, Glycoprotein, Virus, Microglia, Ageing	- Non-replicating models of SARS-CoV-2 can penetrate the BBB and induce neuroinflammation, which is linked to cognitive impairment. - The Delta and Omicron variants of SARS-CoV-2 cross the BBB more rapidly than other variants. - The study indicates a potential risk for individuals with COVID-19, especially Long-COVID, to develop long-term cognitive impairments due to the effects of neuroinflammation.
Qiao et al.	[14]	2024	In Vivo (Mouse, K18-hACE2 hemizygous)	SARS-CoV-2; severe COVID; neuroinflammation; blood–brain barrier; choroid plexus barrier; mouse modelling	- SARS-CoV-2 infection leads to deficiencies in the BBB and the choroid plexus barrier, contributing to neurological complications associated with COVID-19. - The neuroinvasiveness of SARS-CoV-2 remains a critical concern, as the virus can breach barriers in the CNS. - Microvascular damage, pericyte damage, tight junction loss, endothelial activation, and vascular inflammation are identified as the main mechanisms driving BBB impairment. - Cerebrovascular and choroid plexus dysfunctions are factors in the acute phase and persist in long-term neurological effects of COVID-19.

**Table 3:** Main data on blood–brain barrier dysfunction and neurological outcomes in COVID-19 in human cell culture.

Author	Ref.	Year	Study Design	Keywords	Findings
Coelho et al.	[18]	2025	In vitro (Human Brain Microvascular Endothelial Cells—HBMEC)	SARS-CoV-2; endothelial cells; blood–brain barrier; ACE2; des-Arg-bradykinin; inflammation	- SARS-CoV-2 replication in a BBB model leads to increased barrier permeability. - The replication process disables the ACE2-dependent regulation of the bradykinin B1 receptor. - These findings suggest potential implications for understanding the neurological effects of COVID-19. - The authors emphasize the importance of studying the interactions between SARS-CoV-2 and the BBB.
Motta et al.	[17]	2023	In vitro (Human Brain Microvascular Endothelial Cells—HBMEC)	COVID-19; blood–brain barrier; mitochondrial dynamics; NF-κB signalling pathway; endothelial activation	- Exposure to SARS-CoV-2 leads to pro-inflammatory activation in human brain microvascular endothelial cells (HBMECs), even in the absence of active viral replication. - The interaction of host cells with viral surface proteins is sufficient to trigger programmed cell death cascades. - This pro-inflammatory activation is likely mediated by the activation of the non-canonical NF-κB pathway. - The exposure results in mitochondrial and tight junction remodelling, as well as endothelial apoptosis. - The findings suggest that circulating SARS-CoV-2 viral particles or proteins have effects on endothelial cells in the BBB, contributing to a neuroinflammatory state. - These events reflect some aspects of the neurological and vascular manifestations observed in patients with COVID-19.

**Table 4:** Biological source and biomarkers of blood–brain barrier dysfunction and neuroinflammation.

Author	Ref.	Year	Sample Size Biological Source	BBB Markers Assessed	Neuroinflammation/ Damage Markers
Almulla et al.	[24]	2025	Serum (HU) N = 90 (60 Long-COVID 30 Controls)	IgA/IgM antibodies against barrier (e.g., Claudin-5, S100B)	Neural autoantibodies
Atluri, et al.	[27]	2021	N = 11 CSF (HU)	Total Protein (Indirect indicator)	Pleocytosis (cell count)
Boesl et al.	[22]	2024	N = 47 CSF and Serum (HU)	QAlb (CSF albumin quotient)	Total Protein
Bonetto et al.	[15]	2022	N = 77 Plasma and CSF (HU)	S100B (Serum), MMP-9, QAlb	GFAP, NfL, Cytokines (IL-6, IL-8)
Chaganti et al.	[16]	2025	Imaging (MRI) (HU) N = 20 (10 PASC, 10 Controls)	Vascular permeability (Ktrans), DTI-ALPS	-
Coelho et al.	[18]	2025	-Cell Culture (HBMECs) (CC/HU)	TEER (Transendothelial Electrical Resistance), Permeability Assay	Bradykinin Receptor (B1R)
Erickson et al.	[19]	2023	-Brain Tissue (MS)	Fibrinogen and IgG leakage	Iba-1 (Microglia), GFAP (Astrocytes)
Etter et al.	[23]	2022	N = 28 CSF and Plasma (HU)	QAlb	Cytokines (IL-6, IL-10), Autoantibodies
Guasp et al.	[28]	2022	N = 48 CSF (HU)	QAlb	14-3-3 Protein, NfL
Jarius et al.	[21]	2022	N = 127 CSF (HU)	QAlb, Total Protein	Oligoclonal Bands, Intrathecal IgG synthesis
Motta et al.	[17]	2023	-Cell Culture (HBMECs) (CC/HU)	Tight Junction Remodelling	NF-kB Pathway, Cellular Apoptosis
Qiao et al.	[14]	2024	-Brain Tissue and Serum (MS)	Albumin/Fibrinogen Leakage, Claudin-5	CD45+ Cells, Glial Activation
Spatola et al.	[25]	2023	N = 32 CSF and Plasma (HU)	-	Antibody Response (Immunological Imprinting)
Wallensten et al.	[26]	2024	Plasma (HU) N = 315 (Initial) 95 (Follow-up)	S100B, GFAP (Astrocytic/BBB injury markers)	NfL, GDF-15
Zingaropoli et al.	[20]	2022	N = 41 Serum and CSF (HU)	MMP-9 (Metalloproteinase), Estimated BBB integrity	NfL

**Note:** In column 5 (biological source), HU means human, MS means mouse, and CC means cell culture. Samples from cell culture have two mentions, one from the cellular model of the material and the other, HU or MS, from the origin of the cells.

## Data Availability

No new data were created or analyzed in this study. Data sharing is not applicable to this article.

## References

[1] Johnson K.D., Harris C., Cain J.K., Hummer C., Goyal H., Perisetti A. (2020). Pulmonary and Extra-Pulmonary Clinical Manifestations of COVID-19. Front. Med..

[2] Buja L.M., Wolf D.A., Zhao B., Akkanti B., McDonald M., Lelenwa L., Reilly N., Ottaviani G., Elghetany M.T., Trujillo D.O. (2020). The Emerging Spectrum of Cardiopulmonary Pathology of the Coronavirus Disease 2019 (COVID-19): Report of 3 Autopsies from Houston, Texas, and Review of Autopsy Findings from Other United States Cities. Cardiovasc. Pathol..

[3] Huang C., Wang Y., Li X., Ren L., Zhao J., Hu Y., Zhang L., Fan G., Xu J., Gu X. (2020). Clinical Features of Patients Infected with 2019 Novel Coronavirus in Wuhan, China. Lancet.

[4] Nalbandian A., Sehgal K., Gupta A., Madhavan M.V., McGroder C., Stevens J.S., Cook J.R., Nordvig A.S., Shalev D., Sehrawat T.S. (2021). Post-acute COVID-19 syndrome. Nat. Med..

[5] Lau W.L., Zuckerman J.E., Gupta A., Kalantar-Zadeh K. (2021). The COVID-Kidney Controversy: Can SARS-CoV-2 Cause Direct Renal Infection?. Nephron.

[6] Furman S., Green K., Lane T.E. (2024). COVID-19 and the impact on Alzheimer’s disease pathology. J. Neurochem..

[7] Parotto M., Gyöngyösi M., Howe K., Myatra S.N., Ranzani O., Shankar-Hari M., Herridge M.S. (2023). Post-Acute Sequelae of COVID-19: Understanding and Addressing the Burden of Multisystem Manifestations. Lancet Respir. Med..

[8] Nägele M.P., Haubner B., Tanner F.C., Ruschitzka F., Flammer A.J. (2020). Endothelial Dysfunction in COVID-19: Current Findings and Therapeutic Implications. Atherosclerosis.

[9] Perico L., Benigni A., Casiraghi F., Ng L.F.P., Renia L., Remuzzi G. (2021). Immunity, Endothelial Injury and Complement-Induced Coagulopathy in COVID-19. Nat. Rev. Nephrol..

[10] Office for National Statistics (ONS) The Prevalence of Long COVID Symptoms and COVID-19 Complications-Office for National Statistics. https://www.ons.gov.uk/news/statementsandletters/theprevalenceoflongcovidsymptomsandcovid19complications.

[11] Klein R.S. (2022). Mechanisms of Coronavirus Infectious Disease 2019-Related Neurologic Diseases. Curr. Opin. Neurol..

[12] Vallée A. (2021). Dysautonomia and Implications for Anosmia in Long COVID-19 Disease. J. Clin. Med..

[13] A Clinical Case Definition of Post COVID-19 Condition by a Delphi Consensus, 6 October 2021. https://www.who.int/publications/i/item/WHO-2019-nCoV-Post_COVID-19_condition-Clinical_case_definition-2021.1.

[14] Qiao H., Deng X., Qiu L., Qu Y., Chiu Y., Chen F., Xia S., Muenzel C., Ge T., Zhang Z. (2024). SARS-CoV-2 Induces Blood-Brain Barrier and Choroid Plexus Barrier Impairments and Vascular Inflammation in Mice. J. Med. Virol..

[15] Bonetto V., Pasetto L., Lisi I., Carbonara M., Zangari R., Ferrari E., Punzi V., Luotti S., Bottino N., Biagianti B. (2022). Markers of Blood-Brain Barrier Disruption Increase Early and Persistently in COVID-19 Patients with Neurological Manifestations. Front. Immunol..

[16] Chaganti J.R., Talekar T.K., Brew B.J. (2025). Asymmetrical Glymphatic Dysfunction in Patients with Long Covid Associated Neurocognitive Impairment- Correlation with BBB Disruption. BMC Neurol..

[17] Motta C.S., Torices S., da Rosa B.G., Marcos A.C., Alvarez-Rosa L., Siqueira M., Moreno-Rodriguez T., Matos A.d.R., Caetano B.C., Martins J.S.C. (2023). Human Brain Microvascular Endothelial Cells Exposure to SARS-CoV-2 Leads to Inflammatory Activation through NF-κB Non-Canonical Pathway and Mitochondrial Remodeling. Viruses.

[18] Coelho S.V.A., Souza G.L.E., Bezerra B.B., Lima L.R., Correa I.A., De Almeida D.V., Silva-Aguiar R.P.D., Pinheiro A.A.S., Sirois P., Caruso-Neves C. (2025). SARS-Cov-2 Replication in a Blood–Brain Barrier Model Established with Human Brain Microvascular Endothelial Cells Induces Permeability and Disables ACE2-Dependent Regulation of Bradykinin B1 Receptor. Int. J. Mol. Sci..

[19] Erickson M.A., Logsdon A.F., Rhea E.M., Hansen K.M., Holden S.J., Banks W.A., Smith J.L., German C., Farr S.A., Morley J.E. (2023). Blood-Brain Barrier Penetration of Non-Replicating SARS-CoV-2 and S1 Variants of Concern Induce Neuroinflammation Which Is Accentuated in a Mouse Model of Alzheimer’s Disease. Brain Behav. Immun..

[20] Zingaropoli M.A., Iannetta M., Piermatteo L., Pasculli P., Latronico T., Mazzuti L., Campogiani L., Duca L., Ferraguti G., De Michele M. (2022). Neuro-Axonal Damage and Alteration of Blood-Brain Barrier Integrity in COVID-19 Patients. Cells.

[21] Jarius S., Pache F., Körtvelyessy P., Jelčić I., Stettner M., Franciotta D., Keller E., Neumann B., Ringelstein M., Senel M. (2022). Cerebrospinal Fluid Findings in COVID-19: A Multicenter Study of 150 Lumbar Punctures in 127 Patients. J. Neuroinflamm..

[22] Boesl F., Goereci Y., Gerhard A., Bremer B., Raeder V., Schweitzer F., Hoppmann U., Behrens J., Bellmann-Strobl J., Paul F. (2024). Cerebrospinal Fluid Findings in Patients with Neurological Manifestations in Post-COVID-19 Syndrome. J. Neurol..

[23] Etter M.M., Martins T.A., Kulsvehagen L., Pössnecker E., Duchemin W., Hogan S., Sanabria-Diaz G., Müller J., Chiappini A., Rychen J. (2022). Severe Neuro-COVID Is Associated with Peripheral Immune Signatures, Autoimmunity and Neurodegeneration: A Prospective Cross-Sectional Study. Nat. Commun..

[24] Almulla A.F., Maes M., Zhou B., Al-Hakeim H.K., Vojdani A. (2025). Brain-Targeted Autoimmunity Is Strongly Associated with Long COVID and Its Chronic Fatigue Syndrome as Well as Its Affective Symptoms. J. Adv. Res..

[25] Spatola M., Nziza N., Jung W., Deng Y., Yuan D., Dinoto A., Bozzetti S., Chiodega V., Ferrari S., Lauffenburger D.A. (2023). Neurologic Sequelae of COVID-19 Are Determined by Immunologic Imprinting from Previous Coronaviruses. Brain.

[26] Wallensten J., Havervall S., Power Y., Åsberg M., Borg K., Nager A., Thålin C., Mobarrez F. (2024). Oneyear Longitudinal Study on Biomarkers of Blood-Brain Barrier Permeability in COVID-19 Patients. Sci. Rep..

[27] Atluri P., Vasireddy D., Malayala S. (2021). COVID-19 Encephalopathy in Adults. Cureus.

[28] Guasp M., Muñoz-Sánchez G., Martínez-Hernández E., Santana D., Carbayo Á., Naranjo L., Bolós U., Framil M., Saiz A., Balasa M. (2022). CSF Biomarkers in COVID-19 Associated Encephalopathy and Encephalitis Predict Long-Term Outcome. Front. Immunol..

[29] Sharma S., Jagadeesh H., Saxena A., Chakravarthy H., Devanathan V. (2021). Central Nervous System as a Target of Novel Coronavirus Infections: Potential Routes of Entry and Pathogenic Mechanisms. J. Biosci..

[30] Yang R.-C., Huang K., Zhang H.-P., Li L., Zhang Y.-F., Tan C., Chen H.-C., Jin M.-L., Wang X.-R. (2022). SARS-CoV-2 Productively Infects Human Brain Microvascular Endothelial Cells. J. Neuroinflamm..

[31] Karamyan V.T. (2021). Between Two Storms, Vasoactive Peptides or Bradykinin Underlie Severity of COVID-19?. Physiol. Rep..

[32] Theoharides T.C., Conti P. (2021). Be Aware of SARS-CoV-2 Spike Protein: There Is More than Meets the Eye. J. Biol. Regul. Homeost. Agents.

[33] Fekete M., Lehoczki A., Szappanos Á., Toth A., Mahdi M., Sótonyi P., Benyó Z., Yabluchanskiy A., Tarantini S., Ungvari Z. (2025). Cerebromicrovascular Mechanisms Contributing to Long COVID: Implications for Neurocognitive Health. Geroscience.

[34] Buzhdygan T.P., DeOre B.J., Baldwin-Leclair A., Bullock T.A., McGary H.M., Khan J.A., Razmpour R., Hale J.F., Galie P.A., Potula R. (2020). The SARS-CoV-2 Spike Protein Alters Barrier Function in 2D Static and 3D Microfluidic in-Vitro Models of the Human Blood–Brain Barrier. Neurobiol. Dis..

[35] DeOre B.J., Tran K.A., Andrews A.M., Ramirez S.H., Galie P.A. (2021). SARS-CoV-2 Spike Protein Disrupts Blood–Brain Barrier Integrity via RhoA Activation. J. Neuroimmune Pharmacol..

[36] Magro C.M., Mulvey J., Kubiak J., Mikhail S., Suster D., Crowson A.N., Laurence J., Nuovo G. (2021). Severe COVID-19: A Multifaceted Viral Vasculopathy Syndrome. Ann. Diagn. Pathol..

[37] Montezano A.C., Camargo L.L., Mary S., Neves K.B., Rios F.J., Stein R., Lopes R.A., Beattie W., Thomson J., Herder V. (2023). SARS-CoV-2 Spike Protein Induces Endothelial Inflammation via ACE2 Independently of Viral Replication. Sci. Rep..

[38] Martins-Gonçalves R., Hottz E.D., Bozza P.T. (2023). Acute to Post-Acute COVID-19 Thromboinflammation Persistence: Mechanisms and Potential Consequences. Curr. Res. Immunol..

[39] Ryu J.K., Yan Z., Montano M., Sozmen E.G., Dixit K., Suryawanshi R.K., Matsui Y., Helmy E., Kaushal P., Makanani S.K. (2024). Fibrin Drives Thromboinflammation and Neuropathology in COVID-19. Nature.

[40] Nuovo G.J., Magro C., Shaffer T., Awad H., Suster D., Mikhail S., He B., Michaille J.-J., Liechty B., Tili E. (2021). Endothelial Cell Damage Is the Central Part of COVID-19 and a Mouse Model Induced by Injection of the S1 Subunit of the Spike Protein. Ann. Diagn. Pathol..

[41] Leng A., Shah M., Ahmad S.A., Premraj L., Wildi K., Li Bassi G., Pardo C.A., Choi A., Cho S.-M. (2023). Pathogenesis Underlying Neurological Manifestations of Long COVID Syndrome and Potential Therapeutics. Cells.

[42] Fujimoto Y., Abe H., Eiro T., Tsugawa S., Tanaka M., Hatano M., Nakajima W., Ichijo S., Arisawa T., Takada Y. (2025). Systemic Increase of AMPA Receptors Associated with Cognitive Impairment of Long COVID. Brain Commun..

[43] Theoharides T.C., Kempuraj D. (2023). Role of SARS-CoV-2 Spike-Protein-Induced Activation of Microglia and Mast Cells in the Pathogenesis of Neuro-COVID. Cells.

[44] Sfera A., Rahman L., Zapata-Martín Del Campo C.M., Kozlakidis Z. (2023). Long COVID as a Tauopathy: Of “Brain Fog” and “Fusogen Storms”. Int. J. Mol. Sci..

[45] Kırmızıbekmez A.M., Önder A., Özdemir M.Y., Eryiğit Ö.Y., Yurdakoş E. (2025). Is Neurodegeneration Accelerated? Investigating COVID-19’s Impact on Dementia via Functional Connectivity. Noro Psikiyatr. Ars..

[46] Saikarthik J., Saraswathi I., Alarifi A., Al-Atram A.A., Mickeymaray S., Paramasivam A., Shaikh S., Jeraud M., Alothaim A.S. (2022). Role of Neuroinflammation Mediated Potential Alterations in Adult Neurogenesis as a Factor for Neuropsychiatric Symptoms in Post-Acute COVID-19 Syndrome-A Narrative Review. PeerJ.

[47] Shariff S., Uwishema O., Mizero J., Devi Thambi V., Nazir A., Mahmoud A., Kaushik I., Khayat S., Yusif Maigoro A., Awde S. (2023). Long-Term Cognitive Dysfunction after the COVID-19 Pandemic: A Narrative Review. Ann. Med. Surg..

[48] Reas E.T., Alderson-Myers A., Solders S.K., Shen Q., Rivera C.S., Wang X., Stiver J., Banks S.J., Graves J.S. (2025). Long COVID-Related Blood-Brain Barrier Breakdown and Microstructure in Older Adults Are Modified by Sex and Alzheimer’s Disease Genetic Risk. Imaging Neurosci..

[49] Katasonov A.B. (2025). Therapeutic potential of quercetin and its derivatives against COVID-19. Zhurnal Nevrol. Psikhiatrii Im. SS Korsakova.

[50] Watanabe H., Shima S., Mizutani Y., Ueda A., Ito M. (2022). Long COVID: Pathogenesis and Therapeutic Approach. Brain Nerve.

[51] Rubin L.H., Shi W., Azola A., Bhattacharyya A., Dastgheyb R.M., Wu J., Penna C.D., Parker H., Santiuste I., Ehrenspeck A. (2025). Blood-Brain Barrier Disruption in Long COVID and Cognitive Correlates: A Cross-Sectional MRI Study. Brain Behav. Immun..

[52] El-Maradny Y.A., Rubio-Casillas A., Mohamed K.I., Uversky V.N., Redwan E.M. (2023). Intrinsic Factors behind long-COVID: II. SARS-CoV-2, Extracellular Vesicles, and Neurological Disorders. J. Cell. Biochem..

[53] García-Grimshaw M., Sankowski R., Valdés-Ferrer S.I. (2022). Neurocognitive and Psychiatric Post-Coronavirus Disease 2019 Conditions: Pathogenic Insights of Brain Dysfunction Following Severe Acute Respiratory Syndrome Coronavirus 2 Infection. Curr. Opin. Neurol..

[54] Li J., Xu S., Guo S. (2025). Exploring SARS-CoV-2 Impact on Blood-Brain Barrier and Its Composition: A Review. Medicine.

[55] Yin P., Zhang D., Li B., Lei G., Fu X. (2025). Clinical, Thyroid Metabolic, and Inflammatory Features in Pediatric Patients with Post-Acute COVID-19 Neuropsychiatric Symptoms. BMC Infect. Dis..

[56] Fradkin T., Schmidt-Kastner R. (2025). A Data-Mining Analysis of Host Solute Carrier Family Proteins in SARS-CoV-2 Infection with Reference to Brain Endothelial Cells and the Blood-Brain Barrier in COVID-19. Front. Neurol..

[57] Mayer M.G., Fischer T. (2025). Shared Mechanisms of Blood-Brain Barrier Dysfunction and Neuroinflammation in Coronavirus Disease 2019 and Alzheimer Disease. Am. J. Pathol..

[58] Shrestha B.K., Sujakhu E., Karale S., Telagarapu V.M.L. (2025). COVID-19 in Patients with Multiple Sclerosis—A Narrative Review. Mult. Scler. Relat. Disord..

[59] Papadopoulou P., Polissidis A., Kythreoti G., Sagnou M., Stefanatou A., Theoharides T.C. (2024). Anti-Inflammatory and Neuroprotective Polyphenols Derived from the European Olive Tree, Olea Europaea, L., in Long COVID and Other Conditions Involving Cognitive Impairment. Int. J. Mol. Sci..

[60] Wellford S.A., Chen C.-W., Vukovic M., Batich K.A., Lin E., Shalek A.K., Ordovas-Montanes J., Moseman A.P., Moseman E.A. (2024). Distinct Olfactory Mucosal Macrophage Populations Mediate Neuronal Maintenance and Pathogen Defense. Mucosal. Immunol..

[61] Li D., Zhang X., Lu Y., Jing L., Hu H., Song Y., Wu S., Zhu W. (2024). Post-Sepsis Psychiatric Disorder: Pathophysiology, Prevention, and Treatment. Neurol. Sci..

[62] Govednik T., Lainšček D., Kuhar U., Lachish M., Janežič S., Štrbenc M., Krapež U., Jerala R., Atlas D., Manček-Keber M. (2024). TXM Peptides Inhibit SARS-CoV-2 Infection, Syncytia Formation, and Lower Inflammatory Consequences. Antivir. Res..

[63] Siebert H.-C., Eckert T., Bhunia A., Klatte N., Mohri M., Siebert S., Kozarova A., Hudson J.W., Zhang R., Zhang N. (2023). Blood pH Analysis in Combination with Molecular Medical Tools in Relation to COVID-19 Symptoms. Biomedicines.

[64] Camacho R.C., Alabed S., Zhou H., Chang S.L. (2021). Network Meta-Analysis on the Changes of Amyloid Precursor Protein Expression Following SARS-CoV-2 Infection. J. Neuroimmune Pharmacol..

[65] Mullaguri N., Sivakumar S., Battineni A., Anand S., Vanderwerf J. (2021). COVID-19 Related Acute Hemorrhagic Necrotizing Encephalitis: A Report of Two Cases and Literature Review. Cureus.

[66] El-Demerdash A., Metwaly A.M., Hassan A., Abd El-Aziz T.M., Elkaeed E.B., Eissa I.H., Arafa R.K., Stockand J.D. (2021). Comprehensive Virtual Screening of the Antiviral Potentialities of Marine Polycyclic Guanidine Alkaloids against SARS-CoV-2 (COVID-19). Biomolecules.

[67] Nobile B., Durand M., Olié E., Guillaume S., Molès J.P., Haffen E., Courtet P. (2021). The Anti-Inflammatory Effect of the Tricyclic Antidepressant Clomipramine and Its High Penetration in the Brain Might Be Useful to Prevent the Psychiatric Consequences of SARS-CoV-2 Infection. Front. Pharmacol..

[68] Remsik J., Wilcox J.A., Babady N.E., McMillen T.A., Vachha B.A., Halpern N.A., Dhawan V., Rosenblum M., Iacobuzio-Donahue C.A., Avila E.K. (2021). Inflammatory Leptomeningeal Cytokines Mediate COVID-19 Neurologic Symptoms in Cancer Patients. Cancer Cell.

[69] Carossino M., Montanaro P., O’Connell A., Kenney D., Gertje H., Grosz K., Ericsson M., Huber B.R., Subramaniam S., Kirkland T.A. (2021). Fatal Neuroinvasion and SARS-CoV-2 Tropism in K18-hACE2 Mice Is Partially Independent on hACE2 Expression. bioRxiv.

[70] Pantelis C., Jayaram M., Hannan A.J., Wesselingh R., Nithianantharajah J., Wannan C.M., Syeda W.T., Choy K.C., Zantomio D., Christopoulos A. (2021). Neurological, Neuropsychiatric and Neurodevelopmental Complications of COVID-19. Aust. N. Z. J. Psychiatry.

[71] Butsabong T., Felippe M., Campagnolo P., Maringer K. (2021). The Emerging Role of Perivascular Cells (Pericytes) in Viral Pathogenesis. J. Gen. Virol..

[72] Menezes F., Palmeira J.d.F., Oliveira J.D.S., Argañaraz G.A., Soares C.R.J., Nóbrega O.T., Ribeiro B.M., Argañaraz E.R. (2024). Unraveling the SARS-CoV-2 Spike Protein Long-Term Effect on Neuro-PASC. Front. Cell Neurosci..

[73] Afrisham R., Jadidi Y., Davoudi M., Moayedi K., Karami S., Sadegh-Nejadi S., Ashtary-Larky D., Seyyedebrahimi S., Alizadeh S. (2022). Renal, Cardiac, Neurological, Cutaneous and Coagulopathic Long-Term Manifestations of COVID-19 after Recovery; A Review. Epidemiol. Infect..

[74] Ahmad S.J., Feigen C.M., Vazquez J.P., Kobets A.J., Altschul D.J. (2022). Neurological Sequelae of COVID-19. J. Integr. Neurosci..

[75] Alam S.B., Willows S., Kulka M., Sandhu J.K. (2020). Severe Acute Respiratory Syndrome Coronavirus 2 May Be an Underappreciated Pathogen of the Central Nervous System. Eur. J. Neurol..

[76] Almutairi M.M., Sivandzade F., Albekairi T.H., Alqahtani F., Cucullo L. (2021). Neuroinflammation and Its Impact on the Pathogenesis of COVID-19. Front. Med..

[77] Baalbaki N., Slob E.M.A., Kazer S.W., Abdel-Aziz M.I., Bogaard H.J., Golebski K., Maitland-van der Zee A.H. (2025). The Omics Landscape of Long COVID-A Comprehensive Systematic Review to Advance Biomarker, Target and Drug Discovery. Allergy.

[78] Balcom E.F., Nath A., Power C. (2021). Acute and Chronic Neurological Disorders in COVID-19: Potential Mechanisms of Disease. Brain.

[79] Bhardwaj A., Mishra H.P., Goel A., Gupta A. (2023). COVID-19—A Potential Trigger for MOGAD-Associated Optic Neuritis: A Case Report and Literature Review. Ther. Adv. Ophthalmol..

[80] Bommarito G., Garibotto V., Frisoni G.B., Assal F., Lalive P.H., Allali G. (2022). The Two-Way Route between Delirium Disorder and Dementia: Insights from COVID-19. Neurodegener. Dis..

[81] Bougakov D., Podell K., Goldberg E. (2021). Multiple Neuroinvasive Pathways in COVID-19. Mol. Neurobiol..

[82] Burks S.M., Rosas-Hernandez H., Alejandro Ramirez-Lee M., Cuevas E., Talpos J.C. (2021). Can SARS-CoV-2 Infect the Central Nervous System via the Olfactory Bulb or the Blood-Brain Barrier?. Brain Behav. Immun..

[83] Cárdenas G., Fragoso G., Sciutto E. (2022). Neuroinflammation in Severe Acute Respiratory Syndrome Coronavirus-2 (SARS-CoV-2) Infection: Pathogenesis and Clinical Manifestations. Curr. Opin. Pharmacol..

[84] Chakravarty N., Senthilnathan T., Paiola S., Gyani P., Castillo Cario S., Urena E., Jeysankar A., Jeysankar P., Ignatius Irudayam J., Natesan Subramanian S. (2021). Neurological Pathophysiology of SARS-CoV-2 and Pandemic Potential RNA Viruses: A Comparative 102181. Analysis. FEBS Lett..

[85] Chang H., Chen E., Hu Y., Wu L., Deng L., Ye-Lehmann S., Mao X., Zhu T., Liu J., Chen C. (2024). Extracellular Vesicles: The Invisible Heroes and Villains of COVID-19 Central Neuropathology. Adv. Sci..

[86] Chen T.-B., Chang C.-M., Yang C.-C., Tsai I.-J., Wei C.-Y., Yang H.-W., Yang C.-P. (2023). Neuroimmunological Effect of Vitamin D on Neuropsychiatric Long COVID Syndrome: A Review. Nutrients.

[87] Costanza A., Amerio A., Aguglia A., Rossi M., Parise A., Magnani L., Serafini G., Amore M., Martins D., Nguyen K.D. (2024). Reactive Astrocytosis-A Potential Contributor to Increased Suicide in Long COVID-19 Patients?. Brain Sci..

[88] Denaro C.A., Haloush Y.I., Hsiao S.Y., Orgera J.J., Osorio T., Riggs L.M., Sassaman J.W., Williams S.A., Monte Carlo A.R., Da Costa R.T. (2022). COVID-19 and Neurodegeneration: The Mitochondrial Connection. Aging Cell.

[89] Dameche K., Shams S., AlMesallam M.S. (2025). Herpes Zoster (HZ) Over the Past 10 Years: A Systematic Review on Trends, Triggers, and Post-COVID-19 Impact. Cureus.

[90] El-Sayed A., Aleya L., Kamel M. (2021). COVID-19: A New Emerging Respiratory Disease from the Neurological Perspective. Env. Sci. Pollut. Res. Int..

[91] ElBini Dhouib I. (2021). Does Coronaviruses Induce Neurodegenerative Diseases? A Systematic Review on the Neurotropism and Neuroinvasion of SARS-CoV-2. Drug Discov. Ther..

[92] Elechi K.W., Oyepeju Nkem O., Timothy Chibueze N., Elechi U.S., Franklin Chimaobi K. (2025). Long-Term Neurological Consequences of COVID-19 in Patients with Pre-Existing Alzheimer’s and Parkinson’s Disease: A Comprehensive Review. Neurosci. Insights.

[93] Elizalde-Díaz J.P., Miranda-Narváez C.L., Martínez-Lazcano J.C., Martínez-Martínez E. (2022). The Relationship between Chronic Immune Response and Neurodegenerative Damage in Long COVID-19. Front. Immunol..

[94] Filippatos F., Tatsi E.-B., Michos A. (2022). Post-COVID-19 Syndrome in Children (Review). Exp. Ther. Med..

[95] Rourke L., Damant R. (2025). A short version of the post-COVID-19 condition stigma questionnaire. Public Health Pract..

[96] Ghosh S., Das Sarma J. (2025). The Age-Dependent Neuroglial Interaction with Peripheral Immune Cells in Coronavirus-Induced Neuroinflammation with a Special Emphasis on COVID-19. Biogerontology.

[97] Hachem M. (2021). SARS-CoV-2 Journey to the Brain with a Focus on Potential Role of Docosahexaenoic Acid Bioactive Lipid Mediators. Biochimie.

[98] Hashimoto K. (2023). Overview of the Potential Use of Fluvoxamine for COVID-19 and Long COVID. Discov. Ment. Health.

[99] Hein Z.M., Thazin, Kumar S., Che Ramli M.D., Che Mohd Nassir C.M.N. (2025). Immunomodulatory Mechanisms Underlying Neurological Manifestations in Long COVID: Implications for Immune-Mediated Neurodegeneration. Int. J. Mol. Sci..

[100] Jakhmola S., Indari O., Chatterjee S., Jha H.C. (2020). SARS-CoV-2, an Underestimated Pathogen of the Nervous System. SN Compr. Clin. Med..

[101] Kumar R., Harilal S., Sabitha M., Pappachan L.K., Roshni P.R., Mathew B. (2022). Current Perspective of COVID-19 on Neurology: A Mechanistic Insight. Comb. Chem. High. Throughput Screen..

[102] Li J., Long X., Zhang Q., Fang X., Fang F., Lv X., Zhang D., Sun Y., Li N., Hu S. (2021). Emerging Evidence for Neuropsycho-Consequences of COVID-19. Curr. Neuropharmacol..

[103] Yan F., Jin Q., Li Y., Wang X., Dong C., Xia X., Gao Y., Zhang J., Hou Z. (2026). Impact of prolonged infection on SARS-CoV-2 evolution. Microbiol. Spectr..

[104] Li X., Edén A., Malwade S., Cunningham J.L., Bergquist J., Weidenfors J.A., Sellgren C.M., Engberg G., Piehl F., Gisslen M. (2025). Central and Peripheral Kynurenine Pathway Metabolites in COVID-19: Implications for Neurological and Immunological Responses. Brain Behav. Immun..

[105] López Lloreda C. (2024). COVID’s Toll on the Brain: New Clues Emerge. Nature.

[106] Lyra E., Silva N.M., Barros-Aragão F.G.Q., De Felice F.G., Ferreira S.T. (2022). Inflammation at the Crossroads of COVID-19, Cognitive Deficits and Depression. Neuropharmacology.

[107] Méndez-García L.A., Carrillo-Ruiz J.D., Solleiro-Villavicencio H. (2023). Editorial: Short and Long-Term Sequelae within the Central Nervous System Due to COVID-19. Front. Cell. Neurosci..

[108] Monje M., Iwasaki A. (2022). The Neurobiology of Long COVID. Neuron.

[109] Nagu P., Parashar A., Behl T., Mehta V. (2021). CNS Implications of COVID-19: A Comprehensive Review. Rev. Neurosci..

[110] Oldfield P.R., Hibberd J., Bridle B.W. (2021). How Does Severe Acute Respiratory Syndrome-Coronavirus-2 Affect the Brain and Its Implications for the Vaccines Currently in Use. Vaccines.

[111] Østergaard L. (2021). SARS CoV-2 Related Microvascular Damage and Symptoms during and after COVID-19: Consequences of Capillary Transit-Time Changes, Tissue Hypoxia and Inflammation. Physiol. Rep..

[112] Ostermann P.N., Schaal H. (2023). Human Brain Organoids to Explore SARS-CoV-2-Induced Effects on the Central Nervous System. Rev. Med. Virol..

[113] Paradiso B., Limback C., Su T., Liao W., Mpotsaris A. (2023). Editorial: An Update on Neurological Disorders Post COVID-19 Infection. Front. Neurol..

[114] Paris G.C., Azevedo A.A., Ferreira A.L., Azevedo Y.M.A., Rainho M.A., Oliveira G.P., Silva K.R., Cortez E.A.C., Stumbo A.C., Carvalho S.N. (2021). Therapeutic Potential of Mesenchymal Stem Cells in Multiple Organs Affected by COVID-19. Life Sci..

[115] Park S.O., Nanda N. (2025). Long COVID: A Systematic Review of Preventive Strategies. Infect. Dis. Rep..

[116] Plummer A.M., Matos Y.L., Lin H.C., Ryman S.G., Birg A., Quinn D.K., Parada A.N., Vakhtin A.A. (2023). Gut-Brain Pathogenesis of Post-Acute COVID-19 Neurocognitive Symptoms. Front. Neurosci..

[117] Popa E., Popa A.E., Poroch M., Poroch V., Ungureanu M.I., Slanina A.M., Bacusca A., Coman E.A. (2025). The Molecular Mechanisms of Cognitive Dysfunction in Long COVID: A Narrative Review. Int. J. Mol. Sci..

[118] Priyal, Sehgal V., Kapila S., Taneja R., Mehmi P., Gulati N. (2023). Review of Neurological Manifestations of SARS-CoV-2. Cureus.

[119] Raza M.L., Imam M.H., Zehra W., Jamil S. (2024). Neuro-Inflammatory Pathways in COVID-19-Induced Central Nervous System Injury: Implications for Prevention and Treatment Strategies. Exp. Neurol..

[120] Reiss A.B., Greene C., Dayaramani C., Rauchman S.H., Stecker M.M., De Leon J., Pinkhasov A. (2023). Long COVID, the Brain, Nerves, and Cognitive Function. Neurol. Int..

[121] Ribeiro D.E., Oliveira-Giacomelli Á., Glaser T., Arnaud-Sampaio V.F., Andrejew R., Dieckmann L., Baranova J., Lameu C., Ratajczak M.Z., Ulrich H. (2021). Hyperactivation of P2X7 Receptors as a Culprit of COVID-19 Neuropathology. Mol. Psychiatry.

[122] Riederer P., Ter Meulen V. (2020). Coronaviruses: A Challenge of Today and a Call for Extended Human Postmortem Brain Analyses. J. Neural Transm..

[123] Rousseau B.A., Bhaduri-McIntosh S. (2023). Inflammation and Epstein-Barr Virus at the Crossroads of Multiple Sclerosis and Post-Acute Sequelae of COVID-19 Infection. Viruses.

[124] Satoh T., Trudler D., Oh C.-K., Lipton S.A. (2022). Potential Therapeutic Use of the Rosemary Diterpene Carnosic Acid for Alzheimer’s Disease, Parkinson’s Disease, and Long-COVID through NRF2 Activation to Counteract the NLRP3 Inflammasome. Antioxidants.

[125] Shabani Z., Liu J., Su H. (2023). Vascular Dysfunctions Contribute to the Long-Term Cognitive Deficits Following COVID-19. Biology.

[126] Singh D., Singh E. (2022). An Overview of the Neurological Aspects in COVID-19 Infection. J. Chem. Neuroanat..

[127] Smith M.M., Melrose J. (2024). Impaired Instructive and Protective Barrier Functions of the Endothelial Cell Glycocalyx Pericellular Matrix Is Impacted in COVID-19 Disease. J. Cell. Mol. Med..

[128] Suprewicz Ł., Fiedoruk K., Czarnowska A., Sadowski M., Strzelecka A., Galie P.A., Janmey P.A., Kułakowska A., Bucki R. (2023). Blood-Brain Barrier Function in Response to SARS-CoV-2 and Its Spike Protein. Neurol. Neurochir. Pol..

[129] Talkington G.M., Kolluru P., Gressett T.E., Ismael S., Meenakshi U., Acquarone M., Solch-Ottaiano R.J., White A., Ouvrier B., Paré K. (2024). Neurological Sequelae of Long COVID: A Comprehensive Review of Diagnostic Imaging, Underlying Mechanisms, and Potential Therapeutics. Front. Neurol..

[130] Tanwar M., Singh A., Singh T.P., Sharma S., Sharma P. (2024). Comprehensive Review on the Virulence Factors and Therapeutic Strategies with the Aid of Artificial Intelligence against Mucormycosis. ACS Infect. Dis..

[131] Tate W., Walker M., Sweetman E., Helliwell A., Peppercorn K., Edgar C., Blair A., Chatterjee A. (2022). Molecular Mechanisms of Neuroinflammation in ME/CFS and Long COVID to Sustain Disease and Promote Relapses. Front. Neurol..

[132] Theoharides T.C. (2022). Could SARS-CoV-2 Spike Protein Be Responsible for Long-COVID Syndrome?. Mol. Neurobiol..

[133] Theoharides T.C., Klimas N.G. (2024). COVID-19 and Long COVID: Disruption of the Neurovascular Unit, Blood-Brain Barrier, and Tight Junctions. Neuroscientist.

[134] Treviranus G.R.S. (2020). Psychoses by Attacks from Subverted Mast Cells: A Role for Arterial Intramural Flow Badly Steered by the Nasal Ganglia?. Psychiatr. Danub..

[135] Treviranus G.R.S. (2021). The Pandemic‘s Unresolved Infectious Psycho-Neuro-Immunologies: Myeloid Cells, Vessels, Nasal Cross-Roads. Psychiatr. Danub..

[136] Vlaicu S.I., Tatomir A., Cuevas J., Rus V., Rus H. (2023). COVID, Complement, and the Brain. Front. Immunol..

[137] Wang M., Wang J., Ren Y., Lu L., Xiong W., Li L., Xu S., Tang M., Yuan Y., Xie Y. (2024). Current Clinical Findings of Acute Neurological Syndromes after SARS-CoV-2 Infection. MedComm.

[138] Whitmore H.A.B., Kim L.A. (2021). Understanding the Role of Blood Vessels in the Neurologic Manifestations of Coronavirus Disease 2019 (COVID-19). Am. J. Pathol..

[139] Wu X., Xiang M., Jing H., Wang C., Novakovic V.A., Shi J. (2024). Damage to Endothelial Barriers and Its Contribution to Long COVID. Angiogenesis.

[140] Yachou Y., El Idrissi A., Belapasov V., Ait Benali S. (2020). Neuroinvasion, Neurotropic, and Neuroinflammatory Events of SARS-CoV-2: Understanding the Neurological Manifestations in COVID-19 Patients. Neurol. Sci..

[141] Ylikoski J., Lehtimäki J., Pääkkönen R., Mäkitie A. (2022). Prevention and Treatment of Life-Threatening COVID-19 May Be Possible with Oxygen Treatment. Life.

[142] Zubair A.S., McAlpine L.S., Gardin T., Farhadian S., Kuruvilla D.E., Spudich S. (2020). Neuropathogenesis and Neurologic Manifestations of the Coronaviruses in the Age of Coronavirus Disease 2019: A Review. JAMA Neurol..

[143] Łoniewski I., Skonieczna-Żydecka K., Sołek-Pastuszka J., Marlicz W. (2022). Probiotics in the Management of Mental and Gastrointestinal Post-COVID Symptomes. J. Clin. Med..

